# Metabolomics in Biotic Stress: Insights into Potato Resistance to Powdery Scab

**DOI:** 10.3390/plants15081242

**Published:** 2026-04-17

**Authors:** Sadegh Balotf, Richard Wilson, Calum R. Wilson

**Affiliations:** 1 Centre for Crop Health, University of Southern Queensland, Toowoomba, QLD 4350, Australia; 2 Central Science Laboratory, University of Tasmania, Hobart, TAS 7005, Australia; richard.wilson@utas.edu.au; 3 Tasmanian Institute of Agriculture, University of Tasmania, New Town, TAS 7008, Australia

**Keywords:** metabolomics, powdery scab, potato, glutathione metabolism

## Abstract

Powdery scab, caused by *Spongospora subterranea*, is a major disease of potato in which host resistance remains poorly understood at the biochemical level. While previous transcriptomic and proteomic studies have implicated glutathione S-transferases (GSTs) in cultivar-specific defence responses, orthogonal evidence at the metabolite level remains limited. In this study, untargeted metabolomics was applied to investigate root metabolic responses of two potato cultivars with contrasting resistance to *S. subterranea*. The relatively resistant cultivar ‘Gladiator’ and the susceptible cultivar ‘Iwa’ were inoculated with *S. subterranea*, and roots were collected at the stage of visible gall formation for analysis by high-resolution liquid chromatography–mass spectrometry (LC-MS/MS). Principal component analysis revealed a distinct metabolic profile in infected ‘Gladiator’ roots compared with both non-inoculated controls and infected ‘Iwa’ roots, indicating a stronger host metabolic response in the resistant cultivar. Among the annotated metabolites, cysteinyl-glycine (Cys-Gly), a central intermediate of glutathione turnover, was significantly more abundant in infected ‘Gladiator’ roots. The accumulation of Cys-Gly provides direct biochemical evidence linking enhanced glutathione cycling and GST activity to effective host defence. These findings highlight glutathione metabolism as a key component of potato resistance and demonstrate the value of metabolomics for additional validation of biochemical mechanisms underlying plant cultivar responses to biotic stress.

## 1. Introduction

Powdery scab is a widespread and persistent disease affecting potato roots and tubers in major potato-growing regions. The pathogen produces highly durable resting spores that can survive in soil for decades, complicating crop rotation and soil management strategies [1]. Infection results in gall formation on roots and characteristic lesions on tubers, which reduce both yield and market quality [2]. Conventional management strategies, including chemical treatments and cultural practices, have limited efficacy, and the use of resistant cultivars remains the most practical method for long-term control. Some research has explored potential resistance mechanisms and management strategies. For example, increased salicylic acid (SA) levels and enhanced SA signalling have been reported to reduce pathogen proliferation, highlighting SA’s role in induced resistance [3]. Similarly, engineered *Bacillus subtilis* strains delivering immune-eliciting molecules, such as the phytocytokine StPep1, have been shown to decrease root galling and tuber lesions, suggesting a potential biocontrol strategy [4]. Although these preliminary findings are promising, cultivars with strong and reliable resistance remain unavailable, and the molecular and biochemical mechanisms underpinning resistance are still poorly understood, emphasising the need for further research to uncover pathways that could be manipulated by plant breeders to improve crop protection.

Our previous studies used transcriptomics and proteomics to investigate cultivar-specific responses to infection. We observed that sets of glutathione S-transferase (GST) genes and proteins were consistently upregulated in the resistant cultivar ‘Gladiator’, whereas the susceptible cultivar ‘Iwa’ showed minimal induction [5]. This GST-mediated response suggested a key role in detoxifying reactive oxygen species generated during infection, thereby limiting pathogen proliferation. Genome-wide mapping further revealed that potato chromosome nine contains a locus enriched in GST genes associated with quantitative resistance [6].

Recent advances in metabolomics have provided further insight into the biochemical responses of potato cultivars to *S. subterranea* infection. Previous studies identified 24 organic compounds in potato root exudates, including amino acids, sugars, and organic acids, which stimulate resting spore germination [7]. Follow-up work demonstrated that environmental factors such as root vigour, cultivar and nutrient availability influence the composition of root exudates and, consequently, the release of metabolites that activate spore germination [8]. Untargeted metabolomics comparing ten potato cultivars, either susceptible or tolerant to powdery scab, revealed that tolerant cultivars had higher levels of metabolites related to fatty acids, amino acids, phenolics, and cell wall compounds [9]. Barsalote [10] later explored the role of root exudates in disease development, particularly focusing on the amino acids L-glutamine and tyrosine, which were shown to act as stimulants for *S. subterranea* spore germination. The study also investigated how introducing rhizosphere bacteria could modify the root exudate profile to reduce pathogen activation [11].

Plant–pathogen interactions are inherently complex, involving coordinated responses across multiple molecular and biochemical layers. A comprehensive understanding of host resistance, therefore, requires the integration of complementary analytical approaches [12]. In our previous studies, transcriptomic and proteomic analyses were used to identify candidate genes and proteins associated with resistance and susceptibility to *S. subterranea* infection in potato, highlighting a potential role for GST pathways. Building on these findings, the present study employed untargeted metabolomics to examine root biochemical changes in a resistant and a susceptible potato cultivar following *S. subterranea* infection. In particular, we focused on metabolites associated with glutathione metabolism and GST activity, hypothesising that resistant plants would accumulate glutathione-related intermediates, whereas susceptible plants would not.

## 2. Results and Discussion

### 2.1. Distinct Metabolic Profiles in Resistant and Susceptible Cultivars

**Figure 1:**
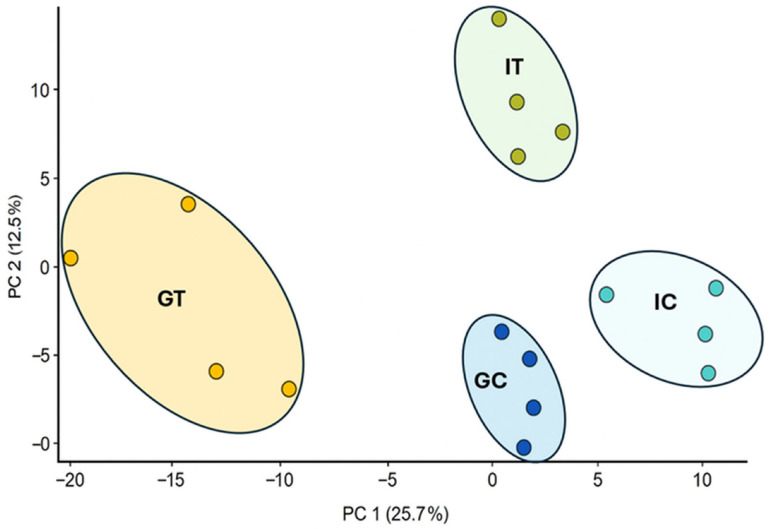
PCA plot showing separation of infected and control samples for ‘Gladiator’ and ‘Iwa’ cultivars. The samples were analysed from four biological replicates, with one plant per replicate. IC: ‘Iwa’ control; IT: ‘Iwa’ treated (infected); GC: ‘Gladiator’ control; GT: ‘Gladiator’ treated.

Principal component analysis (PCA) demonstrated clear separation among the four sample groups along the principal components (Figure 1), reflecting distinct and cultivar-specific metabolic responses to infection. Infected roots of the resistant cultivar ‘Gladiator’ formed a distinct cluster from both its control and the control/treated susceptible cultivar ‘Iwa’, indicating a more pronounced and coordinated metabolic reprogramming in ‘Gladiator’ following pathogen challenge, consistent with previous reports that resistant potato cultivars undergo pronounced metabolic shifts to limit pathogen proliferation [9]. This separation is largely driven by differential accumulation of metabolites with established roles in plant defence, including Cys-Gly, which reflects enhanced glutathione turnover and redox regulation [13]; salicylic acid, a key signalling molecule in systemic acquired resistance; and pipecolic acid, a known amplifier of defence responses. Additional metabolites, such as scopoletin and fraxetin, function as antimicrobial coumarins, while nicotinic acid contributes to NAD/NADP-mediated redox homeostasis, collectively supporting enhanced detoxification and oxidative stress management (Supplementary Table S1). These metabolite-level changes suggest that resistance in ‘Gladiator’ involves coordinated activation of multiple defence pathways, including redox regulation, antimicrobial metabolite production, and systemic defence signalling, whereas the susceptible cultivar exhibits comparatively limited metabolic shifts, consistent with a weaker defence response.

### 2.2. Cysteinyl-Glycine as a Marker of GST-Mediated Resistance

**Figure 2:**
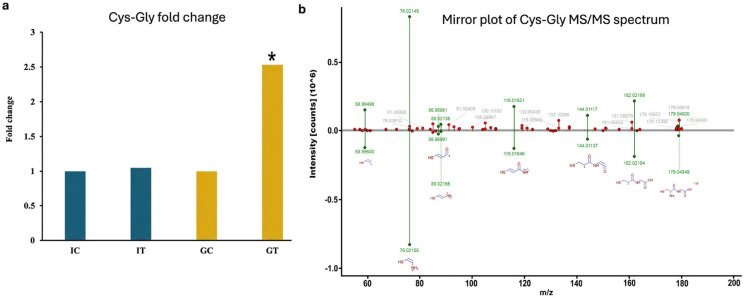
(**a**) Relative peak intensity of Cys-Gly in resistant and susceptible cultivars, showing a significant increase in ‘Gladiator’ upon infection (adjusted *p*-value < 0.05; as indicated by *) but no change in ‘Iwa’; IC: ‘Iwa’ control; IT: ‘Iwa’ treated (infected); GC: ‘Gladiator’ control; GT: ‘Gladiator’ treated. (**b**) Mirror plot of Cys-Gly MS/MS acquired (**top**) and reference (**bottom**) fragment ions, annotated with green bars signifying matching fragments.

Cysteinyl-glycine (Cys-Gly) was significantly more abundant in infected ‘Gladiator’ roots compared with all other groups (fold change ~2.5; adjusted *p* < 0.05) (Figure 2a) and was identified with high confidence (mzCloud match score 91.5; Figure 2b, Supplementary Table S1). This elevation of Cys-Gly in the resistant cultivar provides targeted evidence that glutathione metabolism is differentially modulated during *S. subterranea* infection. Glutathione plays a central role in regulating both plant defence responses and metabolic processes [14]. At the biochemical level, GST catalyse the nucleophilic attack of the glutathione thiolate anion on electrophilic centres of xenobiotic or pathogen-derived compounds, forming glutathione conjugates. These conjugates are subsequently processed by γ-glutamyl transpeptidase and dipeptidases, releasing Cys-Gly as a stable dipeptide intermediate in the degradation pathway. An increase in Cys-Gly thus reflects not only enhanced glutathione turnover but also the upstream formation and processing of glutathione conjugates, a hallmark of elevated GST-mediated detoxification activity under biotic stress [15].

Proper regulation of the Cys-Gly level is potentially important for the prevention of oxidative stress and redox signalling [16,17]. Cys-Gly, as a low-molecular-weight thiol derivative, participates in thiol–disulfide exchange reactions and can influence the cellular glutathione redox couple (GSH/GSSG), thereby contributing to the control of intracellular ROS levels. This function is particularly relevant during pathogen challenge, where transient ROS production acts as both a defence signal and a source of oxidative challenge. Additionally, glutathione is involved in various key functions, including the modulation of signalling pathways, biosynthesis of sulfur-containing metabolites, and the inactivation of harmful compounds [13]. Overall, the specific upregulation of Cys-Gly in ‘Gladiator’, but not in the susceptible cultivar ‘Iwa’, indicates that GST-mediated glutathione turnover and subsequent dipeptide formation are components of an effective resistance response, rather than a general stress response.

**Figure 3:**
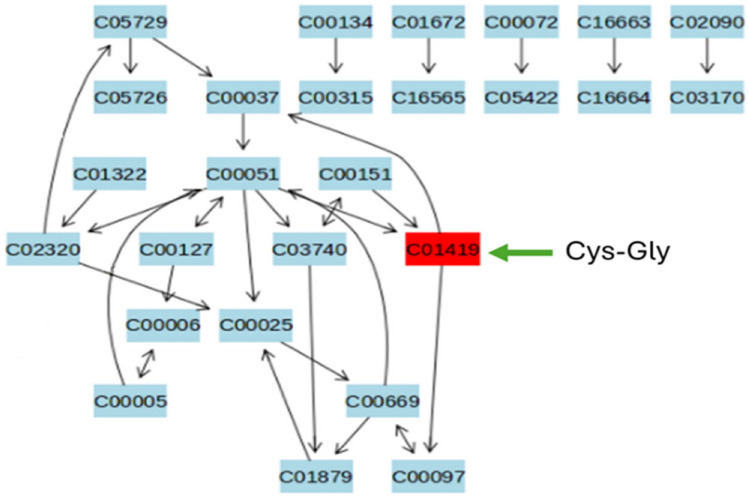
Schematic representation of the glutathione metabolic pathway highlighting cysteinyl-glycine (Cys-Gly) as the only differentially regulated metabolite detected in infected roots of the resistant cultivar ‘Gladiator’.

The identified metabolite was then mapped to the glutathione metabolic pathway (Figure 3). The result shows that only Cys-Gly was differentially regulated in this pathway. The selective accumulation of Cys-Gly in ‘Gladiator’ roots provides direct biochemical evidence of enhanced glutathione cycling and GST-mediated detoxification in the resistant cultivar. Elevated glutathione turnover likely mitigates oxidative stress induced by infection and supports downstream defence signalling [3,4]. As ‘Iwa’ did not exhibit a similar induction, this could limit redox-buffering capacity in the susceptible cultivar. These findings align with prior transcriptomic and proteomic studies demonstrating upregulation of GST genes in resistant cultivars [5,6]. Collectively, the metabolomic data confirm that glutathione metabolism is a key component of potato resistance against powdery scab and may serve as a biochemical marker for breeding or screening resistant cultivars [9].

## 3. Materials and Methods

### 3.1. Plant Material and Inoculum Preparation

The *S. subterranea* sporosori (aggregates of resting spores) were collected from powdery-scab-infected tubers in a commercial field in Tasmania, Australia. Infected tubers were cleaned with tap water and air-dried in a cool, dark place for 1–2 days. The lesions were excised, sifted through a 600 µm sieve, and the sporosori were stored at room temperature in the dark. To release zoospores, three mg of sporosori was incubated in 1.0 mL of Hoagland’s solution. After three days of incubation at 15 °C in the dark, zoospore release was verified by light microscopy. Two potato cultivars, ‘Gladiator’ and ‘Iwa’, were selected for their contrasting resistance to *S. subterranea* infection. The ‘Gladiator’ cultivar is moderately resistant, while ‘Iwa’ is highly susceptible to both root and tuber infections. Potato plants were initially propagated from single-node cuttings and grown in tissue culture under standard conditions using Murashige and Skoog medium supplemented with 500 mg/L casein hydrolysate, 30 g/L sucrose, and 40 mg/L ascorbic acid. After three weeks, plants were removed from the medium and roots were inoculated by suspending them in Hoagland’s solution containing zoospores released from *S. subterranea* sporosori. The inoculated plants were then transplanted into pots with sterilised potting mix and grown in a glasshouse at 25  ±  3 °C and 80  ±  5% relative humidity for six weeks. An additional inoculum of 20 mg dried sporosori was added to each pot 14 days after planting to maintain consistent pathogen pressure. At 42 days post-inoculation, when root galls were visible, roots were harvested, washed, frozen in liquid nitrogen, and stored at −80 °C until further analysis.

### 3.2. Metabolite Extraction and HPLC/MS Analysis

Metabolites and proteins were extracted simultaneously from potato root tissue. Briefly, 50 mg of frozen potato root tissue was homogenised in a protein extraction buffer using a bead beater to ensure thorough cell disruption. Following homogenisation, the extracts were centrifuged at 16,000× *g* for 10 min in a cold room (4 °C). The supernatant was then mixed with six volumes of absolute acetone, precooled to −20 °C, and stored at −20 °C overnight. The samples were centrifuged at 10,000× *g* for 8 min to separate the proteins from the soluble components. The supernatant, which is typically discarded in standard protein extraction protocols, was carefully retained. This supernatant was then used for metabolite analysis, providing a distinct phase for the evaluation of metabolites without the interference of proteins. Extracted metabolites were reconstituted in 2% (*v*/*v*) acetonitrile in HPLC grade water and analysed using a Q-Exactive HF Orbitrap mass spectrometer coupled with an Ultimate 3000 RSLC HPLC system (Thermo Scientific, Waltham, MA, USA). MS detection was carried out in a heated electrospray ionisation (ESI) source in positive ionisation modes using an ESI spray voltages of 3.9 kV, capillary temperature of 320 °C, S-Lens RF of 50 V and sheath gas of 40 AU. Samples were separated on a reversed-phase 2.1 × 150 mm column and guard cartridge with 3 mm C18 silica packing (both Acclaim PolarAdvatage II, Thermo Scientific) held at 35 °C. A 20-min gradient from 98% mobile phase A (water containing 0.1% *v*/*v* formic acid) to 65% mobile phase B (acetonitrile containing 0.1% *v*/*v* formic acid) and HPLC flow rate of 300 mL/min were used. Full scan MS1 spectra were acquired at 120,000 resolution over the m/z range 70–1000 (AGC target of 3 × 10^6^ ions) while MS2 spectra were acquired at 15,000 resolution (AGC target of 1 × 10^5^ ions) using a Top10 data-dependent acquisition method and a stepped normalised collision energy of 15, 35 and 60. Both MS1 and MS2 spectra were acquired in profile mode.

### 3.3. Data Pre-Processing and Visualisation

Raw LC–MS files were processed using Compound Discoverer software (Thermo Scientific, version 3.1) with the default untargeted metabolomics workflow. Compounds were filtered to retain only named metabolites with precursor m/z deviations between −5 and +5 ppm and fully matched MS^2^ spectra against the mzCloud high-resolution spectral library. For two sample test analyses, the data were imported into Perseus v1.6.14.0 [18] and log2-transformed. Metabolites were classified as significantly altered in abundance between groups based on Student’s *t*-test (FDR  <  0.05 with the s0 parameter set to 0.1). Principal component analysis (PCA) was conducted on the filtered dataset comprising 198 annotated metabolites (Supplemental Table S1) to evaluate global metabolic differences among sample groups. Pathway analysis was performed in MetaboAnalyst 6.0 [19] to explore and visualise the associated metabolic pathways. The samples were analysed from four biological replicates (one plant per replicate).

## 4. Conclusions

Advances in omics technologies have provided extensive insights into plant–pathogen interactions; however, for *S. subterranea*, there remains a notable gap in this type of information [20]. The current metabolomic study highlights the functional importance of glutathione metabolism in potato resistance to *S. subterranea*. The observed accumulation of Cys-Gly in the resistant cultivar ‘Gladiator’ suggests that enhanced redox regulation and detoxification are key processes limiting pathogen impact. In addition, salicylic acid accumulated to significantly higher levels (>2-fold) in the resistant cultivar but not in the susceptible cultivar (Supplementary Table S1), indicating the activation of salicylic acid-dependent defence responses specifically associated with resistance. Salicylic acid is a central signalling molecule in plant immunity, known to regulate local and systemic defence responses and to promote resistance against biotrophic and hemibiotrophic pathogens [3,21]; its selective accumulation in the resistant cultivar further supports the presence of a stronger and more effective defence response compared with the susceptible cultivar.

Importantly, GSTs represent a large and functionally heterogeneous multigene family in potato, with approximately 90 members identified in the genome. Our previous transcriptomic and proteomic analyses [5,6] demonstrated that individual GST isoforms exhibit distinct expression profiles under stress conditions, indicating functional divergence rather than redundancy among family members. In this context, basal GST expression and glutathione turnover are expected to occur in the resistant and susceptible cultivars (in both infected and uninfected plants) as part of constitutive redox homeostasis and detoxification processes. However, the present metabolomic data reveal the genotype-dependent modulation of glutathione metabolism, with significantly greater accumulation of Cys-Gly in the resistant cultivar following infection. This differential accumulation suggests enhanced glutathione turnover and conjugation activity, potentially associated with specific GST isoforms that are more actively engaged in the resistant genotype. Collectively, these findings indicate that resistance is likely mediated by the selective activation of specific components of the GST network, rather than a uniform response across the entire gene family, although functional validation is required to resolve the precise roles of individual GSTs.

These results provide a biochemical perspective on resistance, demonstrating how specific metabolites contribute to defence responses and complement broader resistance strategies. Future research could focus on time-resolved targeted metabolomics to capture the dynamics of glutathione-related compounds during infection, employ CRISPR-mediated manipulation of GST genes to directly test their role in resistance, and extend analyses across diverse potato germplasm to determine the consistency of these metabolic responses. Such approaches would not only clarify the mechanistic basis of GST-linked defence but also inform the development of practical tools for screening and breeding powdery scab-resistant cultivars.

## Data Availability

The metabolomics raw data are available via The Metabolomics Workbench https://www.metabolomicsworkbench.org/ (accessed on 12 April 2026) under study ID: ST004422 (https://doi.org/10.21228/M8N26R).

## Supplementary Materials

The following supporting information can be downloaded at: https://www.mdpi.com/article/10.3390/plants15081242/s1, Table S1: List of identified metabolites.

## Abbreviations

The following abbreviations are used in this manuscript:

Cys-Gly	Cysteinyl-Glycine
GST	glutathione S-transferase
LC	Liquid Chromatography
MS	Mass Spectrometry
PCA	Principal Component Analysis
SA	Salicylic Acid
